# 3D intraoral scanner as an auxiliary tool for the diagnosis of dental hard-tissue conditions

**DOI:** 10.1007/s00784-025-06683-w

**Published:** 2025-12-05

**Authors:** Astrid C. Valdivia Tapia, J. Sebastian Lara, E. Angeles Martinez-Mier, Giovanna C. Denucci, Frank Lippert, George J. Eckert, Anderson T. Hara

**Affiliations:** 1 Department of Biomedical and Applied Sciences, Indiana University School of Dentistry, 415 Lansing St, Indianapolis, 46202 IN USA; 2 Department Comprehensive Care and Allied Professions, Indiana University School of Dentistry, Indianapolis, IN USA; 3 Department of Dental Public Health and Dental Informatics, Indiana University School of Dentistry, Indianapolis, IN USA; 4 Department of Biostatistics and Health Data Science, School of Medicine, Indiana University, Indianapolis, IN USA

**Keywords:** 3D Intraoral scanner, Dental caries, Erosive tooth wear, Dental fluorosis

## Abstract

**Aim:**

This in vitro study aimed to compare the direct (visual exam) versus indirect (3D-colored digital images exam) assessments of ICDAS (dental caries), BEWE (erosive tooth wear), and TF (enamel fluorosis lesions) indices performed on the occlusal surfaces of extracted human teeth for the detection and severity differentiation of dental caries, erosive tooth wear, and enamel fluorosis lesions.

**Materials and methods:**

A total of 453 occlusal surfaces from extracted human teeth were mounted on 28 typodont models. Surfaces were examined directly (visual) and indirectly using 3D-coloured intraoral scans (TRIOS 4, 3Shape). Three indices were assessed: International Caries Detection and Assessment System (ICDAS; caries), Basic Erosive Wear Examination (BEWE; erosive tooth wear), and Thylstrup-Fejerskov (TF; fluorosis). Agreement between methods was analysed using weighted kappa statistics and percentage agreement.

**Results:**

Substantial agreement was observed between direct and indirect methods: ICDAS (κ = 0.72, 64%), BEWE (κ = 0.70, 73%), and TF (κ = 0.75, 86%). Early lesions (ICDAS 1–2, BEWE 1) were more frequently misclassified.

**Conclusions:**

3D-coloured intraoral scanner imaging shows substantial agreement with visual examination for detecting and grading caries, erosive tooth wear, and fluorosis.

**Clinical significance:**

This study supports the use of intraoral scanners as a practical adjunct to visual examination for documentation, education, and potentially remote consultations. Further clinical validation is warranted.

## Introduction

Dental hard-tissue conditions, including dental caries, erosive tooth wear (ETW), and enamel fluorosis, are highly prevalent worldwide. Dental caries affects nearly 90% of adults [1], while enamel fluorosis is observed in an estimated 23% of the general population [2]– [3]. ETW impacts approximately 77% [4]. These conditions are not only widespread but can significantly impair individuals’ quality of life [4–7].

Traditionally, the assessment of dental hard-tissue conditions has relied on clinical visual examination and subjective grading systems, particularly in research settings. This approach requires considerable examiner training, repeated calibration, and is inherently dependent on the clinician’s experience and interpretation. Recent advances in digital dentistry particularly the development of high-resolution intraoral scanners (IOS) offer new possibilities for objective and reproducible assessments [8–11].

IOS technology projects structured light onto dental surfaces to produce three-dimensional (3D) colored digital models that capture detailed morphological and surface characteristics [8–13]. Initially developed for restorative dentistry, IOS applications have since expanded into orthodontic, periodontal and surgical fields, where they are primarily used for treatment planning, digital model analysis, and appliance fabrication rather than diagnosis [14]. However, its use as an auxiliary diagnostic tool for detecting and monitoring dental hard-tissue conditions remains underexplored. Most existing studies have investigated individual lesions such as proximal or occlusal caries [15]– [16], early ETW [17], or lesion progression monitoring [18] with limited evidence regarding enamel fluorosis detection [11, 14, 17].

Moreover, previous studies have often focused on posterior occlusal surfaces typically third molars and assessed either advanced or early stages of disease in isolation, depending on the diagnostic index applied [16–21]. Therefore, the broader potential of IOS for simultaneous detection and differentiation of multiple hard-tissue conditions has yet to be fully investigated.

This study aimed to compare direct (clinical visual examination) and indirect (assessment of 3D colored digital images obtained via IOS) evaluations of occlusal surfaces of extracted human teeth using three established indices: ICDAS (International Caries Detection and Assessment System) for dental caries [22], BEWE (Basic Erosive Wear Examination) for erosive tooth wear [23], and TF (Thylstrup and Fejerskov) index for enamel fluorosis [24]. The objective was to evaluate the diagnostic agreement and the ability of IOS to differentiate between conditions and severity levels of caries, ETW, and fluorosis.

## Methods

### Experimental design

A convenience sample of extracted human teeth with or without dental caries, ETW, or enamel fluorosis lesions were collected from an institutional tooth bank, which receives donations from dentists across different regions of the country. Their collection and use were previously approved by the Institutional Review Board of Indiana University (# NS0911-07). The teeth were mounted on typodont models (*n* = 28 models/453 occlusal surfaces) to simulate complete dental arches for scanning and assessment. Teeth were mounted on typodont models by a different operator to avoid prior contact by the examiner and reduce potential bias. A single calibrated examiner in ICDAS (dental caries), BEWE (ETW), and TF (enamel fluorosis lesions) indices performed the direct and indirect visual evaluations. The BEWE index was applied per surface to enable surface-level agreement analysis between methods, which differs from its original sextant-based design. The examiner evaluated the complete typodont model during both visual and digital assessments, rather than isolated teeth, to replicate a clinical full-mouth examination. Kappa statistical analysis was used with a significance level of 0.05 to compare the direct and indirect methods. The study was reported following the STARCARDDS adaptation for dental research [25].

### Typodont models

**Fig. 1:**
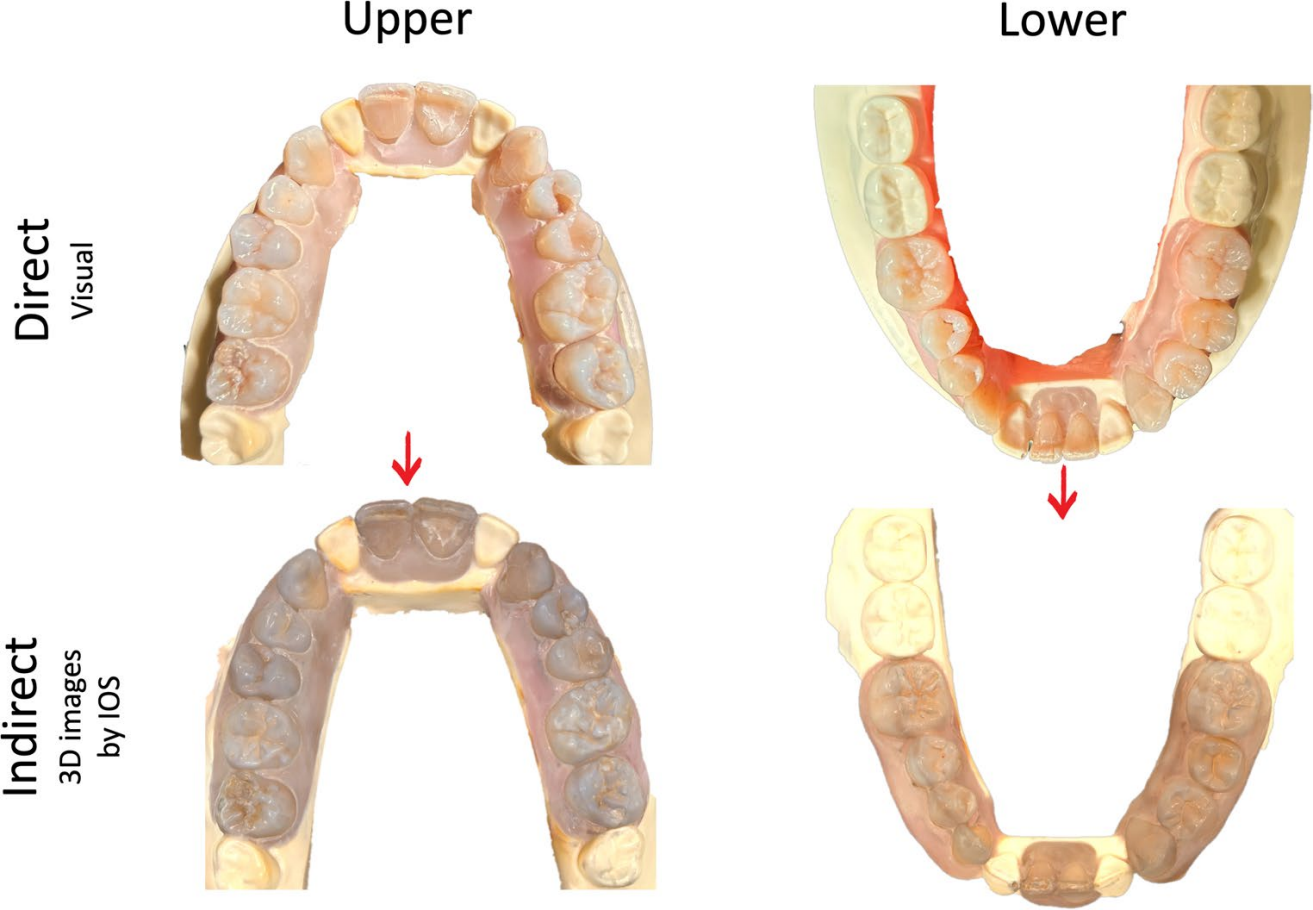
Examples of the upper and lower dental arches observed through the direct method (represented by photos) and the indirect method (3D images generated by IOS)

**Fig. 2:**
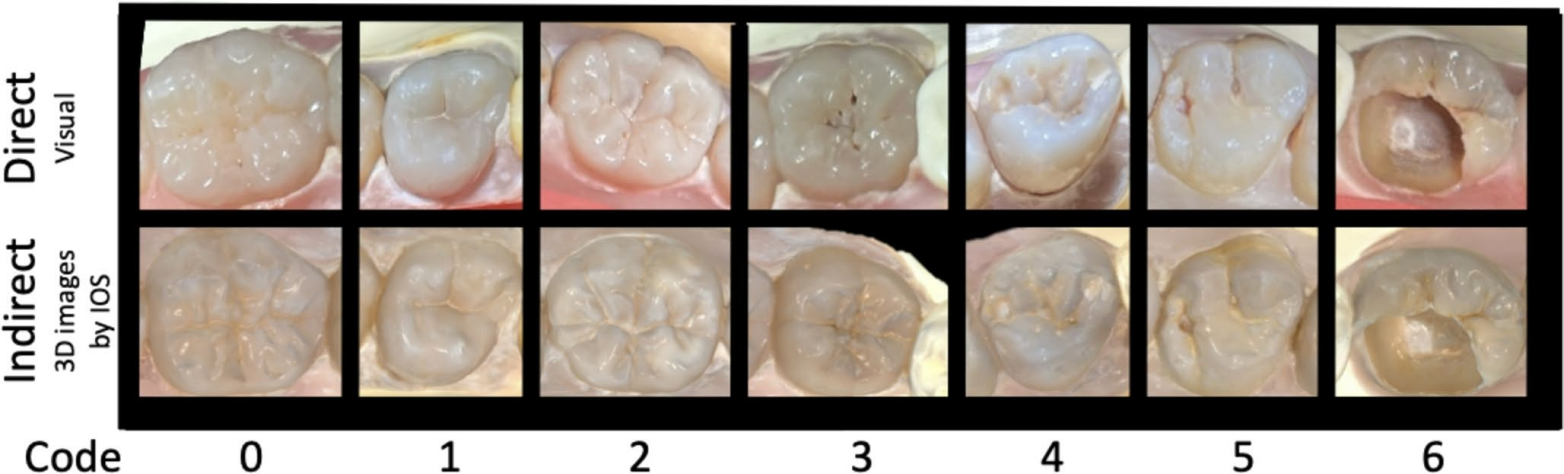
Examples of ICDAS codes observed through the direct method (represented by photos) and the indirect method (3D images generated by IOS)

**Fig. 3:**
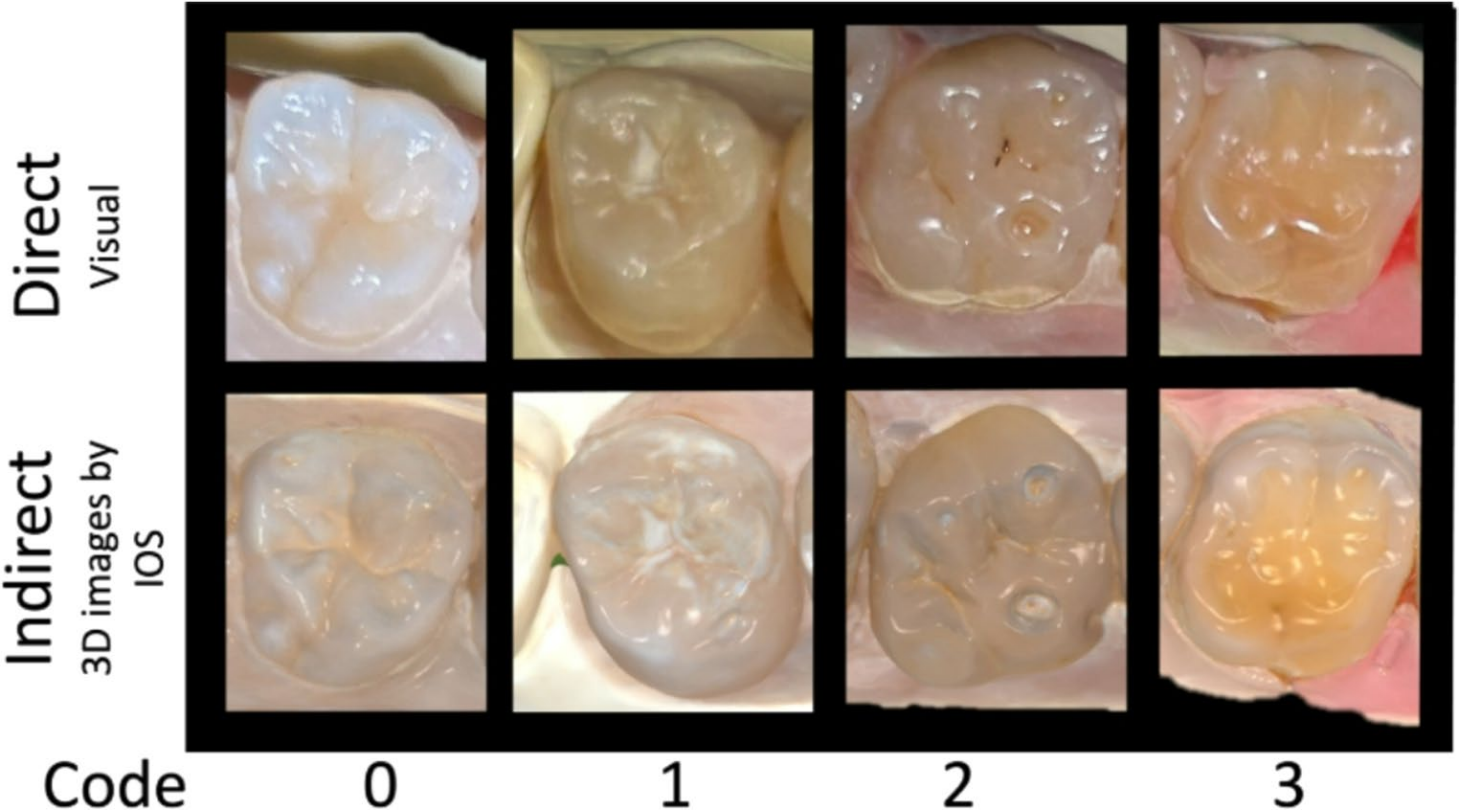
Examples of BEWE codes observed through the direct method (represented by photos) and the indirect method (3D images generated by IOS)

**Fig. 4:**
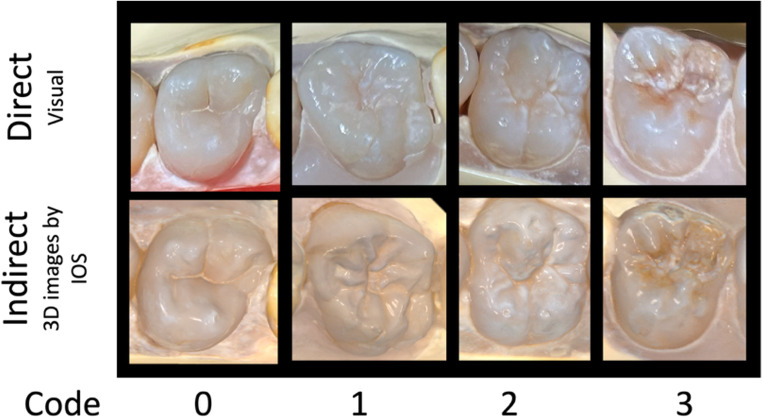
Examples of TF codes observed through the direct method (represented by photos) and the indirect method (3D images generated by IOS)

Human teeth were collected with natural lesions of dental caries, ETW, enamel fluorosis lesions, and sound teeth. The occlusal surface of the selected teeth had different severity codes of each index (ICDAS 0–6; BEWE 0–3 and TF 0–3) (Table 1) and were stored in moist conditions at 4 °C. To simulate the correct anatomical position in the oral cavity, teeth were mounted on typodont models in proper anatomical order, forming upper and lower arches. For this study, *n* = 28 models were assembled, obtaining a total of 453 occlusal surfaces to be analyzed (Fig. 1). Examples of ICDAS, BEWE, and TF codes observed through the direct method (represented by photos) and the indirect method (3D images generated by IOS) ist possible to see in Fig. 2. Figures 3 and 4.

**Table 1:** Dental conditions, indices and severity codes

Condition	Index	Code
None		All index 0
Caries	ICDAS*	1 Initial caries
		2 Distinct visual changes in enamel
		3 Localized enamel breakdowns due to caries with no visible dentine
		4 Underlying dark shadow from dentine
		5 Distinct cavity with visible dentine
		6 Extensive distinct cavity with visible dentine
Erosive Tooth wear	BEWE*	1 Mild erosive tooth wear: Initial loss of surface texture.
		2 Moderate erosive tooth wear: Distinct defect: hard tissue loss involving less than 50% of the surface area.
		3 Severe erosive tooth wear: Hard tissue loss involving more than 50% of the surface area. Moderate and severe levels may involve dentine exposure.
Enamel fluorosis**	TF*	1 Narrow white lines
		2 Pronounced opacity
		3 Cloudy areas of opacity

* *ICDAS* International Caries Detection and Assessment System, *BEWE* Basic Erosion Wear Examination, *TF* Thylstrup-Fejerskov Index

** The other TF scores were not included due to a lack of samples.

### Direct assessment method

A previously trained and calibrated examiner (AV) in the three different indices: ICDAS for caries lesions (weighted kappa = 0.94), BEWE for ETW (weighted kappa = 0.95), and TF for enamel fluorosis lesions (weighted kappa = 0.95) performed the visual analysis. Training and calibration included clinical photographs and extracted teeth, and the examiner’s scores were compared with experts for each index. The excellent agreement observed was likely facilitated by the fact that this was a laboratory-based exercise rather than a clinical one, thereby avoiding common clinical challenges such as limited access to teeth, presence of saliva, mirror fogging, and patient-related factors. Specific conditions were standardized for the study: tooth surface drying using compressed air for ~ 5 s, dental overhead light positioned directly over the teeth, and adequate dental instruments such as a periodontal WHO probe and dental mirror for each index. Data were tabulated and saved on Excel (Microsoft ^®^ Excel for Mac, Version 16.81, 24011420 ©2024 Microsoft).

### Indirect assessment method

The same examiner was previously trained for using the scanner (3Shape 4 intraoral) and dedicated software (3Shape Unite; Version 22.1.1, Copyright © 2023 3Shape A/S) for data processing, according to the manufacturer’s instructions. Scanning conditions were standardized by drying all tooth surfaces with compressed air, and no direct lighting over the sample/teeth to be scanned. The 3D images analysis was also standardized. The entire screen of a 15.5” laptop (Dell, 11th Gen Intel^®^ Core™ i7-11850 H@ 2.50 GHz; Edition Windows 10 Pro) was also used to view each sample. Dental arches were analyzed separately, starting with the upper. The 3D image of each sextant was magnified in order to be seen in full screen, and each tooth surface was evaluated (display resolution 1920 × 1080; landscape, 100% brightness, color space: standard dynamic range). The examiner assigned the score for each index according to what was seen on the screen using the software.

Surfaces with indeterminate scores due to artifacts or poor visibility were excluded; no imputation was used. Missing data were excluded from analysis. The study used a convenience sample of extracted teeth, designed to provide preliminary validation data; therefore, no formal sample size calculation was performed.

### Statistical analysis

Agreement between the direct and indirect methods was assessed using cross-tabulations, kappa statistics, and percent agreement; weights for the weighted kappa calculations were based on the absolute differences between scores (linear weighting). A Kappa of 0.01–0.20 was considered as slight agreement, 0.21–0.40 as fair, 0.41–0.60 as moderate, 0.61–0.80 as substantial, and 0.81–1.00 as almost perfect agreement. Analyses were performed using SAS version 9.4 (SAS Institute, Cary, NC).

## Results

Three hundred ninety-two occlusal surfaces for ICDAS, 352 for BEWE, and 361 for TF index were considered scorable (it was not possible to classify 101 surfaces due to the presence of restorations, fractures, or large ETW for caries, large caries lesions for BEWE or both for enamel fluorosis lesions).

### ICDAS

Table 2 presents the number of surfaces and percentage of agreement between the direct clinical (visual) method and the indirect method using 3D images generated by an intraoral scanner (IOS) for detecting dental caries, classified according to ICDAS codes. The overall weighted kappa was 0.72, with an overall percent agreement of 64%, indicating substantial agreement between direct visual and IOS-based assessments. Sound surfaces (ICDAS 0) showed the highest concordance, with 89 surfaces (66%) classified identically by both methods. Early enamel changes were more prone to misclassification: among surfaces scored as ICDAS 1 clinically, only 22 (42%) were matched by IOS. For ICDAS 2, the IOS correctly identified 68 surfaces (72%). For ICDAS 3, agreement was notably high, with 37 surfaces (88%) scored consistently across methods. In contrast, ICDAS 4 displayed the lowest concordance, with only 7 surface (18%) correctly identified. ICDAS 6 lesions demonstrated excellent agreement. The total overestimation was 22% and underestimation was 14%.

**Table 2:** Number of surfaces (percentage of agreement) according to the ICDAS codes, classified by the direct (visual) and indirect (3D images generated by IOS) methods, n=392. The percentages in parentheses represent the agreement between methods for each specific code. The weighted kappa was calculated using absolute differences between scores (linear weighting)

ICDAS	Clinical (direct method)									Weighted kappa	% agreement
IOS (indirect method)	**Code**	**0**	**1**	**2**	**3**	**4**	**5**	**6**	**Total**	0.72	64
	**0**	**89 (66%)**	12 (23%)	3 (3%)	0 (0%)	0 (0%)	0 (0%)	0 (0%)	104		
	**1**	20 (15%)	**22 (42%)**	6 (6%)	0 (0%)	0 (0%)	0 (0%)	0 (0%)	48		
	**2**	16 (12%)	14 (26%)	**68 (72%)**	2 (5%)	7 (18%)	0 (0%)	0 (0%)	107		
	**3**	9 (7%)	5 (9%)	18 (19%)	**37 (88%)**	23 (59%)	0 (0%)	0 (0%)	92		
	**4**	0 (0%)	0 (0%)	0 (0%)	1 (2%)	**7 (18%)**	0 (0%)	0 (0%)	8		
	**5**	0 (0%)	0 (0%)	0 (0%)	2 (5%)	2 (5%)	**27 (100%)**	0 (0%)	31		
	**6**	0 (0%)	0 (0%)	0 (0%)	0 (0%)	0 (0%)	0 (0%)	**2 (100%)**	2		
	**Total**	134	53	95	42	39	27	2	392		

### BEWE

Table 3. presents the number of surfaces and percentage of agreement for assessing erosive tooth wear, classified according to BEWE codes. The overall weighted kappa was 0.70, with an overall percent agreement of 73%, also indicating substantial agreement. Sound surfaces (BEWE 0) showed the highest concordance, with 144 surfaces (80%) classified consistently. For BEWE 1, representing early wear, only 23 surfaces (43%) were correctly identified. For BEWE 2, the IOS correctly identified 75 surfaces (75%). Severe lesions (BEWE 3) showed strong concordance, with 15 surfaces (83%) scored consistently. The total overestimation was 16%, and the underestimation was 11%.

**Table 3.** Number of surfaces (percentage of agreement) according to the BEWE codes, classified by the direct (visual) and indirect (3D images generated by IOS) methods, n=392. The percentages in parentheses represent the agreement between methods for each specific code. The weighted kappa was calculated using absolute differences between scores (linear weighting)

BEWE	Clinical (direct method)						Weighted kappa	% agreement
IOS (indirect method)	**Code**	**0**	**1**	**2**	**3**	**Total**	0.7	73
	**0**	**144 (80%)**	15 (27%)	3 (3%)	0 (0%)	162		
	**1**	20 (11%)	**23 (43%)**	16 (16%)	0 (0%)	59		
	**2**	16 (9%)	16 (30%)	**75 (75%)**	3 (17%)	110		
	**3**	0 (0%)	0 (0%)	6 (6%)	**15 (83%)**	21		
	**Total**	180	54	100	18	352		

### TF

Table 4 compares both methods across different severity levels, ranging from no fluorosis (TF 0) to mild fluorosis (TF 3). For enamel fluorosis, the overall weighted kappa was 0.75, with an overall percent agreement of 86%, representing substantial agreement. Sound enamel (TF 0) demonstrated excellent concordance, with 261 surfaces (96%) demonstrated agreement. Mild fluorosis showed more variability: for TF 1, only 28 surfaces (53%) were correctly identified. For TF 2, the IOS correctly identified 15 surfaces (56%). Moderate fluorosis (TF 3) was identified with high accuracy, with 6 surfaces (75%) demonstrated classified. The total overestimation was 6%, and the underestimation was 9%.

**Table 4:** Number of surfaces (percentage of agreement) according to the TF codes, classified by the direct (visual) and indirect (3D images generated by IOS) methods, n=392. The percentages in parentheses represent the agreement between methods for each specific code. The weighted kappa was calculated using absolute differences between scores (linear weighting)

TF	Clinical (direct method)						Weighted kappa	% agreement
IOS (indirect method)	**Code**	**0**	**1**	**2**	**3**	**Total**	0.75	86
	**0**	**261 (96%)**	22 (41%)	0 (0%)	0 (0%)	283		
	**1**	11 (4%)	**28 (53%)**	7 (26%)	0 (0%)	46		
	**2**	1 (0%)	3 (6%)	**15 (56%)**	2 (25%)	21		
	**3**	0 (0%)	0 (0%)	5 (18%)	**6 (75%)**	11		
	**Total**	273	53	27	8	361		

## Discussion

This study explored the diagnostic agreement between direct visual examination and indirect assessment using 3D-coloured intraoral scans (IOS) for three validated indices: ICDAS (dental caries), BEWE (erosive tooth wear), and TF (enamel fluorosis) in occlusal surfaces. Overall, IOS demonstrated substantial agreement with visual examination, particularly for sound surfaces and advanced lesions, supporting its potential role as an adjunctive diagnostic tool in preventive and restorative dentistry.

For caries detection using ICDAS, the highest agreement was observed for sound surfaces (ICDAS 0) and cavitated lesions (ICDAS 5–6), where surface breakdown is readily visible. Agreement for ICDAS 3 also remained high (88%). However, early non-cavitated lesions (ICDAS 1–2) were frequently misclassified, typically underestimated as ICDAS 0 or overestimated as ICDAS 3. These discrepancies are consistent with prior reports [15, 17–24, 26] and likely reflect the challenges of detecting subtle demineralization without lesion hydration during scanning, as well as the limited optical sensitivity of current IOS systems to subtle color or translucency changes. The lowest agreement was recorded for ICDAS 4 (18%), which requires the detection of underlying dentinal shadowing, a feature not easily visualized on surface-rendered digital models.

For erosive tooth wear, IOS showed good agreement with BEWE scores, particularly for BEWE 0 and BEWE 3. Moderate agreement was observed for BEWE 2 (75%), whereas BEWE 1, representing early surface texture changes, was the most difficult to detect accurately. Similar limitations in detecting early ETW have been reported in previous studies [17, 27–29], likely due to the fine-scale morphological alterations falling below the current spatial resolution threshold of IOS technology. An interesting finding in this study was that several surfaces clinically scored as BEWE 0 were classified as BEWE 1 or 2 on intraoral scans. This discrepancy may reflect underestimation during clinical inspection due to the inherent limitations of visual examination, such as restricted lighting, angulation, and examiner subjectivity. Intraoral scans, by contrast, provide magnification and allow visualization from multiple perspectives, potentially facilitating the detection of early textural changes that might be overlooked clinically. While this enhanced sensitivity could represent an advantage of digital assessment, further studies are needed to confirm whether these digitally detected changes correspond to clinically relevant erosive wear. This finding may highlight the potential of digital assessment to identify early erosive changes that could benefit from preventive intervention before becoming clinically visible.

Regarding enamel fluorosis, substantial agreement was achieved across most TF scores, especially for sound enamel (TF 0, 96%). Mild fluorosis (TF 1–2) demonstrated lower agreement (53–56%), often being misclassified as adjacent scores. This reflects the inherent dependence of TF scoring on subtle changes in opacity and color, which are highly sensitive to lighting conditions, surface hydration, and scanner color calibration. As this study included only mild fluorosis cases, further research including moderate and severe TF scores is needed to establish diagnostic validity across the full range of severity.

These findings are aligned with existing literature on IOS diagnostic performance for caries and tooth wear [15, 17–24, 26, 29]– [30] and extend current evidence by providing preliminary data on fluorosis detection. Across indices, early lesions (ICDAS 1–2, BEWE 1, TF 1–2) were the most prone to misclassification, reinforcing the need for improvements in IOS resolution, color fidelity, and standardization of acquisition protocols.

Beyond diagnostic performance, IOS offers several clinical advantages. The ability to store, share, and compare 3D images over time supports longitudinal monitoring, interdisciplinary communication, patient education, and tele-consultation. Moreover, intraoral scanning holds potential for broader applications beyond caries, ETW, and fluorosis. Recent studies have demonstrated promising roles in the detection of developmental enamel defects such as molar–incisor hypomineralization (MIH) [31], monitoring gingival inflammation through volumetric and colorimetric changes, and measuring gingival recessions with high reproducibility [32]. These possibilities highlight the broader scope of IOS not only in hard tissue diagnostics but also in soft tissue monitoring. A prior study [11] has also reported reduced chairside time and improved patient comfort. Nevertheless, IOS should not be viewed as a replacement for calibrated visual examination, but rather as a complementary tool that enhances clinical documentation and decision-making.

Some limitations of this study warrant consideration. All assessments were performed on dried extracted teeth, which may have altered the visibility of early lesions. Instead, the sample was designed to ensure the inclusion of teeth with caries, erosive tooth wear, and enamel fluorosis for comparative purposes. This approach may limit the generalizability of the findings to clinical populations. The absence of a histological reference standard may increase the risk of bias; however, this limitation was due to resource constraints and the preservation of teeth for subsequent research. The same examiner performed all assessments, while this approach ensured methodological consistency, we acknowledge that it may have increased the potential for recall bias. The absence of moderate and severe fluorosis cases limits the generalizability of TF-related findings. In addition, typodonts were assembled to simulate complete arches, but paired teeth with symmetrical fluorosis were not available, this limitation does not affect the within-sample comparisons but may reduce representativeness for symmetric conditions such as fluoprisis. Moreover, the results are specific to a single IOS system (TRIOS 4, 3Shape); performance may vary across devices, software versions, and lighting conditions. These factors highlight the importance of future clinical validation and standardization across platforms.

## Conclusion

This in vitro study found substantial agreement between intraoral scanner–based assessments and direct visual examination for caries (ICDAS), erosive tooth wear (BEWE), and enamel fluorosis (TF). IOS was most reliable for sound and advanced lesion scores, while early lesions remained challenging to detect. These findings support the potential of IOS as a valuable adjunctive tool for identifying and monitoring common dental hard-tissue conditions in clinical and educational settings. Integrating IOS into diagnostic protocols may enhance documentation, facilitate remote assessments, and strengthen preventive care strategies.es. Continued technological refinement and clinical validation are needed to strengthen its diagnostic reliability, particularly for early lesions.

## Data Availability

All data generated or analyzed during this study are included in this article. Further enquiries can be directed to the corresponding author.

## References

[1] Borg-Bartolo R, Roccuzzo A, Molinero-Mourelle P et al (2022) Global prevalence of edentulism and dental caries in middle-aged and elderly persons: a systematic review and meta-analysis. J Dent 127:104335. 10.1016/j.jdent.2022.104335 36265526 10.1016/j.jdent.2022.104335

[2] Martignon S, Bartlett D, Manton DJ, Martinez-Mier EA, Splieth C, Avila V (2021) Epidemiology of erosive tooth wear, dental fluorosis and molar incisor hypomineralization in the American continent. Caries Res 55(1):1–11. 10.1159/000512483 33440378 10.1159/000512483

[3] Bartlett D, Dugmore C (2008) Pathological or physiological erosion–is there a relationship to age? Clin Oral Investig 12(Suppl 1):S27–S31. 10.1007/s00784-007-0177-1 18228061 10.1007/s00784-007-0177-1 PMC2238780

[4] Neurath C, Limeback H, Osmunson B, Connett M, Kanter V, Wells CR (2019) Dental fluorosis trends in US oral health surveys: 1986 to 2012. JDR Clin Trans Res 4(4):298–308. 10.1177/2380084419830957 30931722 10.1177/2380084419830957

[5] Zaror C, Matamala-Santander A, Ferrer M, Rivera-Mendoza F, Espinoza-Espinoza G, Martínez-Zapata MJ (2022) Impact of early childhood caries on oral health-related quality of life: a systematic review and meta-analysis. Int J Dent Hyg 20(1):120–135. 10.1111/idh.12494 33825317 10.1111/idh.12494

[6] Di Giovanni T, Eliades T, Papageorgiou SN (2018) Interventions for dental fluorosis: a systematic review. J Esthet Restor Dent 30(6):502–508. 10.1111/jerd.12408 30194793 10.1111/jerd.12408

[7] Stephens MB, Wiedemer JP, Kushner GM (2018) Dental problems in primary care. Am Fam Physician 98(11):654–660 30485039

[8] Mangano F, Gandolfi A, Luongo G, Logozzo S (2017) Intraoral scanners in dentistry: a review of the current literature. BMC Oral Health 17(1):149 Published 2017 Dec 12. 10.1186/s12903-017-0442-x 29233132 10.1186/s12903-017-0442-x PMC5727697

[9] Zimmermann M, Mehl A, Mörmann WH, Reich S (2015) Intraoral scanning systems - a current overview. Int J Comput Dent 18(2):101–129 26110925

[10] Martin CB, Chalmers EV, McIntyre GT, Cochrane H, Mossey PA (2015) Orthodontic scanners: what’s available? J Orthod 42(2):136–143. 10.1179/1465313315Y.0000000001 25939980 10.1179/1465313315Y.0000000001

[11] Suese K (2020) Progress in digital dentistry: the practical use of intraoral scanners. Dent Mater J 39(1):52–56. 10.4012/dmj.2019-224 31723066 10.4012/dmj.2019-224

[12] Ting-Shu S, Jian S (2015) Intraoral digital impression technique: a review. J Prosthodont 24(4):313–321. 10.1111/jopr.12218 25220390 10.1111/jopr.12218

[13] Sonmez N, Gultekin P, Turp V, Akgungor G, Sen D, Mijiritsky E (2018) Evaluation of five CAD/CAM materials by microstructural characterization and mechanical tests: a comparative in vitro study. BMC Oral Health 18(1):5. 10.1186/s12903-017-0458-2 29321010 10.1186/s12903-017-0458-2 PMC5764017

[14] Angelone F, Ponsiglione AM, Ricciardi C, Cesarelli G, Sansone M, Amato F (2023) Diagnostic applications of intraoral scanners: a systematic review. J Imaging 9(7):134. 10.3390/jimaging9070134 37504811 10.3390/jimaging9070134 PMC10381333

[15] Metzger Z, Colson DG, Bown P, Weihard T, Baresel I, Nolting T (2022) Reflected near-infrared light versus bite-wing radiography for the detection of proximal caries: a multicenter prospective clinical study conducted in private practices. J Dent 116:103861. 10.1016/j.jdent.2021.103861 34706269 10.1016/j.jdent.2021.103861

[16] Ntovas P, Michou S, Benetti AR et al (2023) Occlusal caries detection on 3D models obtained with an intraoral scanner. A validation study. J Dent 131:104457. 10.1016/j.jdent.2023.104457 36858167 10.1016/j.jdent.2023.104457

[17] Michou S, Vannahme C, Ekstrand KR, Benetti AR (2020) Detecting early erosive tooth wear using an intraoral scanner system. J Dent 100:103445. 10.1016/j.jdent.2020.103445 32750388 10.1016/j.jdent.2020.103445

[18] Schlenz MA, Schlenz MB, Wöstmann B, Jungert A, Ganss C (2022) Intraoral scanner-based monitoring of tooth wear in young adults: 12-month results. Clin Oral Investig 26(2):1869–1878. 10.1007/s00784-021-04162-6 34498100 10.1007/s00784-021-04162-6 PMC8816769

[19] Sá G, Michou S, Bönecker M, Mendes F, Amarante B, Ekstrand K (2024) Diagnostic validity of ICDAS clinical criteria on digital 3D models. J Dent 149:105274 39084547 10.1016/j.jdent.2024.105274

[20] Jones B, Chen T, Michou S, Kilpatrick N, Burgner DP, Vannahme C, Silva M (2024) Diagnostic agreement between visual examination and an automatedscanner system with fluorescence for detecting and classifying occlusal carious lesions in primary teeth. J Dent 149:105279 39121599 10.1016/j.jdent.2024.105279

[21] Al-Seelawi Z, Hermann NV, Peutzfeldt A, Baram S, Bakke M, Sonnesen L, Tsakanikou A, Rahiotis C, Benetti AR (2024) Clinical and digital assessment of tooth wear. Sci Rep 14(1):592 38182632 10.1038/s41598-023-50107-2 PMC10770026

[22] Pitts NB, Ekstrand KR, ICDAS Foundation (2013) International Caries Detection and Assessment System (ICDAS) and its International Caries Classification and Management System (ICCMS) - methods for staging of the caries process and enabling dentists to manage caries. Community Dent Oral Epidemiol 41(1):e41–e52. 10.1111/cdoe.12025 24916677 10.1111/cdoe.12025

[23] Schlueter N, Amaechi BT, Bartlett D et al (2020) Terminology of erosive tooth wear: consensus report of a workshop organized by the ORCA and the cariology research group of the IADR. Caries Res 54(1):2–6. 10.1159/000503308 31610535 10.1159/000503308

[24] Thylstrup A, Fejerskov O (1978) Clinical appearance of dental fluorosis in permanent teeth in relation to histologic changes. Community Dent Oral Epidemiol 6(6):315–328. 10.1111/j.1600-0528.1978.tb01173.x 282114 10.1111/j.1600-0528.1978.tb01173.x

[25] Neuhaus KW, Eggmann F, Kühnisch J, Kapor S, Janjic Rankovic M, Schüler I, Krause F, Lussi A, Michou S, Ekstrand K, Huysmans MC (2022) STAndard Reporting of CAries Detection and Diagnostic Studies (STARCARDDS). Clin Oral Investig 26(2):1947–1955. 10.1007/s00784-021-04173-3 34623505 10.1007/s00784-021-04173-3 PMC8816754

[26] Schlenz MA, Schupp B, Schmidt A et al (2022) New caries diagnostic tools in intraoral scanners: a comparative in vitro study to established methods in permanent and primary teeth. Sensors 22(6):2156. 10.3390/s22062156 35336328 10.3390/s22062156 PMC8950989

[27] Wulfman C, Koenig V, Mainjot AK (2018) Wear measurement of dental tissues and materials in clinical studies: a systematic review. Dent Mater 34(6):825–850. 10.1016/j.dental.2018.03.002 29627079 10.1016/j.dental.2018.03.002

[28] Kumar S, Keeling A, Osnes C, Bartlett D, O’Toole S (2019) The sensitivity of digital intraoral scanners at measuring early erosive wear. J Dent 81:39–42. 10.1016/j.jdent.2018.12.005 30578831 10.1016/j.jdent.2018.12.005

[29] Marro F, Jacquet W, Martens L, Keeling A, Bartlett D, O’Toole S (2020) Quantifying increased rates of erosive tooth wear progression in the early permanent dentition. J Dent 93:103282. 10.1016/j.jdent.2020.103282 32006669 10.1016/j.jdent.2020.103282

[30] Kanar Ö, Tağtekin D, Korkut B (2024) Accuracy of an intraoral scanner with near-infrared imaging feature in detection of interproximal caries of permanent teeth: an in vivo validation. J Esthet Restor Dent 36(6):845–857. 10.1111/jerd.13198 38263949 10.1111/jerd.13198

[31] Gentile ACC, Marinho GB, Amarante BC, Souza ACMD, Costa VSD, Vilhena FV, Bönecker M (2025) REFIX layer in children with MIH: thickness, color, and hypersensitivity, a preliminary longitudinal study using an intraoral scanner. J Appl Biomater Funct Mater 23:22808000251349931. 10.1177/22808000251349931 40567235 10.1177/22808000251349931

[32] Hassan MA, Silva do Amaral GCL, Saraiva L, Holzhausen M, Mendes FM, Pannuti CM, Stewart B, Malheiros ZM, Benítez C, Nakao LYS, Villar CC, Romito GA (2025) Colorimetric analysis of intraoral scans: a novel approach for detecting gingival inflammation. J Periodontol 96(8):848–857. 10.1002/JPER.24-0389 39826138 10.1002/JPER.24-0389

